# A Not So Common Iliac Vein Anomaly: A Case Report

**DOI:** 10.7759/cureus.38145

**Published:** 2023-04-26

**Authors:** Avery N Love, George A Nasser, Connor Yost

**Affiliations:** 1 Internal Medicine, A.T. Still University-School of Osteopathic Medicine in Arizona (ATSU-SOMA), Mesa, USA; 2 Cardiology, Nasser Cardiology and Vein Center, Woodlands, USA

**Keywords:** anatomy, venography, may-thurner syndrome, duplication, duplicated, common iliac vein

## Abstract

Arising from the external and internal iliac veins, the common iliac veins (CIVs) carry blood from the lower extremities and pelvic region into the inferior vena cava at the level of the fifth lumbar vertebra. It is sometimes common to observe slight anomalies in vascular anatomy in patients; however, anomalies of the CIVs are rare. We present a case of a patient with significant edema of the left lower extremity due to extrinsic compression (May-Thurner syndrome [MTS]) involving a duplicated left CIV found during vascular angiography. Anomalies in pelvic vasculature are well documented in the medical literature; however, documented cases of a duplicated CIV remain few and far between. These anomalies in pelvic vascular anatomy are essential to be aware of to avoid surgical complications and understand their implications in associated pathologies.

## Introduction

The common iliac veins (CIVs) arise from the junction between the external iliac and internal iliac veins. The internal iliac vein drains the lesser pelvis. In contrast, the external iliac vein is continuous with the femoral vein at the level of the inguinal ligament and is responsible for receiving blood flow from the lower extremity. From the junction level between these veins at the sacroiliac joint, the left CIV follows an oblique direction to connect at the inferior vena cava at the fifth lumbar vertebral level, joining the contralateral CIV. The right CIV is vertical, whereas the left CIV is oblique and longer. The left CIV can sometimes ascend to the level of the left renal vein and join at the inferior vena cava anteriorly to the aorta [1-2]. Slight anomalies in vascular anatomy are common, but there are some significant anomalies that are rarer. One anomaly that can occur is duplication, in which either CIV can be doubled in part or throughout its length. The duplicated channel can rejoin at the inferior vena cava, or one channel can join into the contralateral CIV. The anomalies can occur isolated or be associated with other pathologies [3,4]. In one study, it was found that out of 59 cadavers, nine presented with anomalies of the major pelvic veins [5]. Although these anomalies are documented in medical literature, the occurrence of a duplicated CIV is rare. It is important to understand these possible CIV anomalies as they can play a role in certain pathologies. It is also crucial to be aware of these anomalies in performing surgeries as there is a great risk of intraoperative hemorrhage from injury to these vessels [6]. May-Thurner syndrome (MTS) is a condition in which the left CIV is compressed between the right common iliac artery and the lumbar vertebral body, causing physiological changes within the vasculature. MTS was first described in 1957 by May and Thurner when they found this anomaly with intraluminal spurs in the CIV in 22% of 430 cadavers they had autopsied [7]. External compression of the CIV in MTS leads to focal sclerosis and increased risk of venous stasis disease with or without thrombus formation [8].

## Case presentation

**Figure 1:**
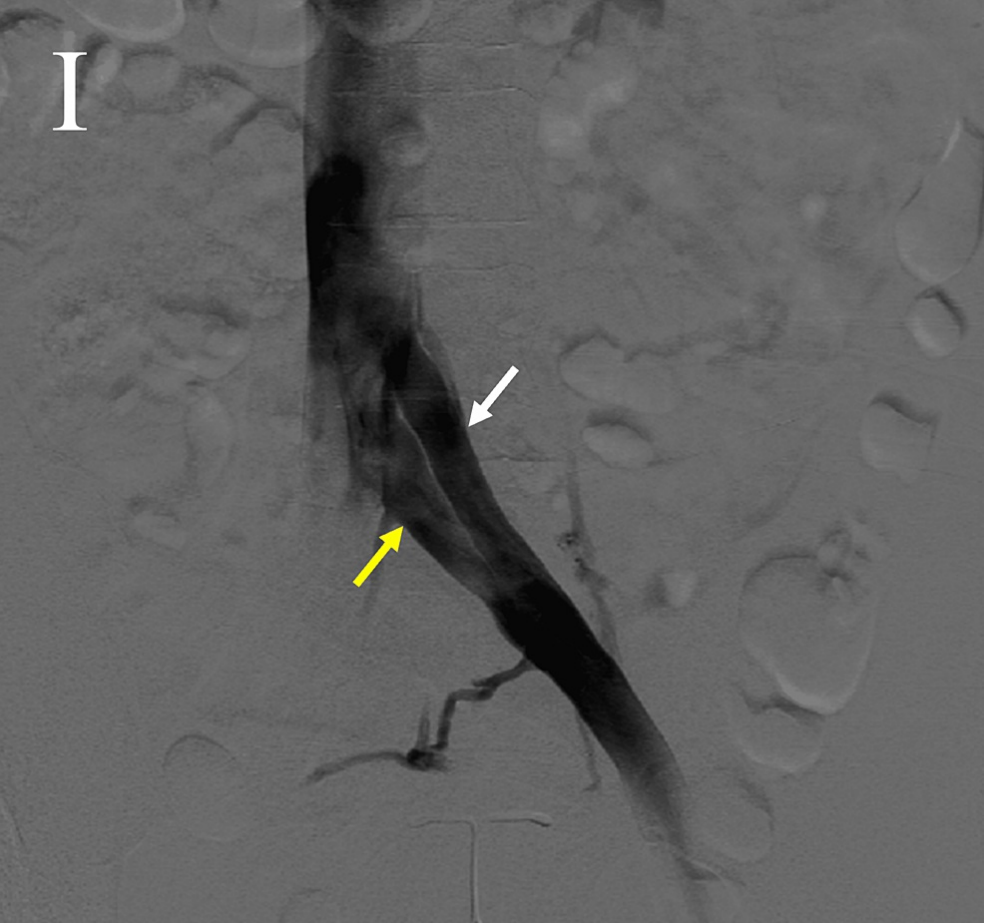
Venography revealing a duplicated left common iliac vein. The white arrow shows the superior branch of the duplicated left common iliac vein, and the yellow arrow shows the inferior branch of the duplicated left common iliac vein.

**Figure 2:**
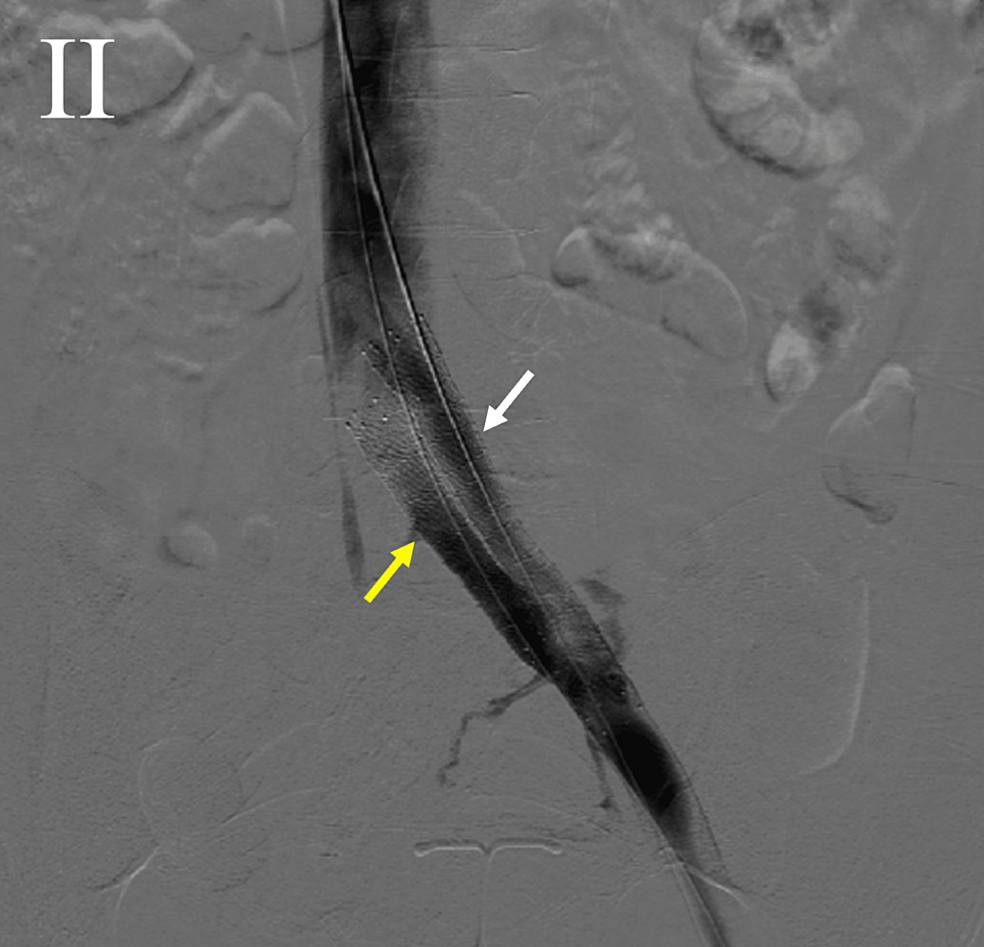
Post-procedural venography revealing patency of both branches of the duplicated left common iliac vein (white arrow: superior branch; yellow arrow: inferior branch).

A 40-year-old female presented to the cardiology clinic in the year 2020. She had a medical history of deep venous thrombosis (DVT) in the left lower extremity (LLE), which occurred in 2001, factor V Leiden, and methylenetetrahydrofolate reductase deficiency. At this time, the patient complained of pain, cramping, and restlessness in the bilateral lower extremities, exacerbated by physical activity, with the LLE being more severe than the right. She reported that these symptoms have been present since her DVT in 2001. The patient also reported that her LLE has chronically been bigger than her right. On the physical exam, her LLE was found to be diffusely larger in circumference than the right, with medial, ropey varicosities (clinical [C], etiological [E], anatomical [A], and pathophysiological [P] [CEAP] Class 2). A reflux study and magnetic resonance venography (MRV) were performed to assess for venous insufficiency and check for MTS, considering the size discrepancy of the LLE to the right. Results of the reflux study revealed venous insufficiency of the bilateral great saphenous veins and the left small saphenous vein. In contrast, the MRV revealed MTS of the distal left CIV. With a confirmed diagnosis of MTS, the patient was then scheduled for venography with possible angioplasty and stenting. The patient was taken to the cardiac catheterization lab (cath lab), and venography revealed a duplicated left CIV (Figure 1). Percutaneous transluminal venoplasty (PTV) was performed on both branches of the duplicated CIV, the superior branch was stented with a Vici 14x90 mm stent, and the inferior branch with a Vici 14x60 mm stent. Kissing balloon inflation of both branches of the left CIV was then done. Venography revealed the patency of both branches at the end of the procedure (Figure 2). The patient was then seen in the office three months later for ultrasound-guided foam sclerotherapy of the left leg and endovenous ablation of the left lesser and greater saphenous veins.

**Figure 3:**
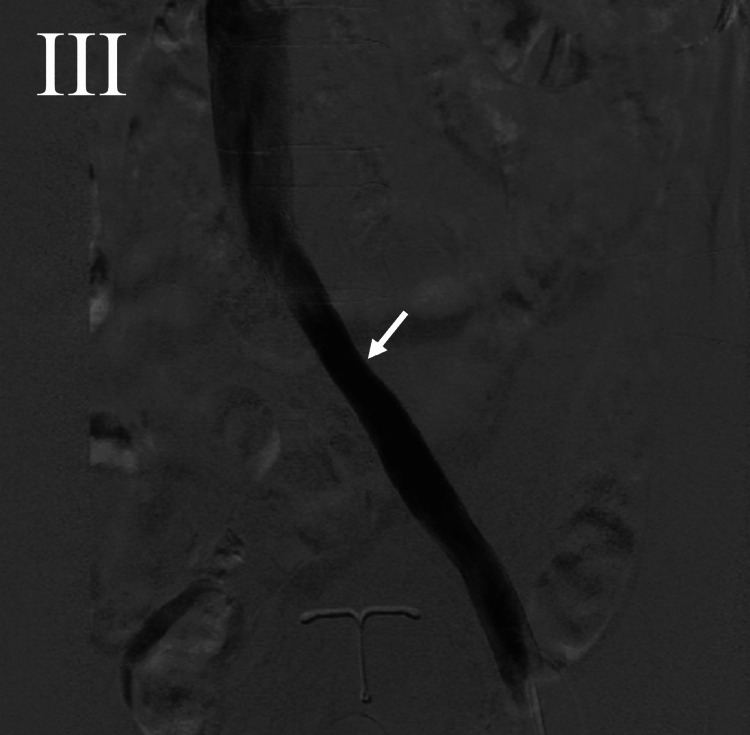
Venography revealing occlusion of the inferior branch of the duplicated left common iliac vein. Only the superior branch remains patent (white arrow).

After the procedure, the patient’s edema resolved until a year later, when she returned to the clinic with a recurrence of the LLE edema. The patient was brought back to the cath lab and was found to have an occlusion of the stent in the inferior branch of the left CIV (Figure 3). Attempts of venoplasty of the inferior branch were unsuccessful. The patients’ edema was then treated with furosemide and conservative management with exercise, elevation, compression stockings, and a lymphedema pump, greatly improving symptoms.

## Discussion

Understanding the embryological development of the vasculature can provide a better understanding of how these anomalies can occur. During week five of embryologic development, the venous system begins to form, giving rise to the first three veins, one of which is the cardinal vein. The cardinal veins are responsible for the drainage of most of the embryo and goes on to form the anterior cardinal veins, which drain the cephalad part of the embryo, and the posterior cardinal veins, which drain the rest of the embryo. During week seven of development, three more veins form, including the sacrocardinal veins, which drain the lower extremities. The anastomosis between the sacrocardinal veins forms the left CIV. The right sacrocardinal vein forms the sacrocardinal segment of the inferior vena cava with which the left CIV anastomoses [9]. Anomalies in the CIVs occur due to maldevelopment of the posterior cardinal veins during embryological development [3]. Duplication, an anomaly of the CIV in which either CIV can be doubled in part or throughout its length, has been reported previously in a few cases. Duplication was first documented by Edwards EA in 1951 at Harvard Medical School as he discussed anomalies he had witnessed in pelvic vasculature during dissections [10]. This anomaly was again reported in 1975 by Sperling M et al., where they observed duplication of the external iliac vein and CIV, with one of the branches of the CIV crossing ventrally to the external iliac vein and common iliac artery [11]. Using computerized tomography in 1998, Meyer DR et al. had a case where they observed duplication of the inferior vena cava involving the CIV [12]. Aside from duplication, Bergman goes on to describe many other cases of different anomalies involving the CIVs [3]. Surgical procedures involving the retroperitoneum are common and therefore necessitate a firm grasp of pelvic vasculature and any possible variations in the anatomy [13,14]. A lack of knowledge of these anomalies increases the risk of vascular injury, a major cause of intraoperative hemorrhage [6]. Not only is it important to be aware of anomalies in pelvic vascular anatomy from a surgical standpoint but also from a medical standpoint, as anomalies in pelvic vasculature can be associated with certain pathologies. One such pathology is Klippel-Trenaunay syndrome, a complex congenital disorder characterized by capillary and venous malformations and limb overgrowth, with or without lymphatic malformations [15]. One study found that in 559 patients with Klippel-Trenaunay syndrome, 19 patients had anomalies in the iliac veins [16].

These CIV anomalies most often appear isolated and may cause edema, venous insufficiency, and thromboembolism. A case report in 2018 reported a patient with a duplicated CIV who developed edema and pain in the LLE following a hysterectomy and was found to have thrombi in the inferior vena cava and iliac vein [17]. It has been found that up to 5-6.7% of adults with spontaneous DVT have an anomaly of the inferior vena cava [18]. Ruggeri M et al. proposed in 2001 that congenital anomalies of the pelvic vasculature may be a risk factor for DVT due to formed collateral veins being unable to withstand an increase in blood flow during physical exertion, thus leading to venous stasis and clotting [19]. The incidence and prevalence of MTS are unknown, but it is believed to be underreported because most patients are asymptomatic and do not require treatment. However, in patients who present with lower extremity venous disorders, MTS is estimated to be the etiology in 2-5% of patients [20]. Symptomatic patients tend to have pain and swelling of the LLE, although involvement in the right and bilateral lower extremities has also been reported [21]. MTS tends to occur in women in the second or third decade of life, with multiple pregnancies, postpartum phases, oral contraceptive pill use, prolonged immobilization, and prolonged durations of dehydration [8]. Diagnosis of MTS requires a high index of suspicion and should be suspected in patients with the risk factors stated previously with unilateral lower extremity swelling. Diagnosis is based on clinical presentation and Doppler; however, MRI, CT, and venography (the gold standard for diagnosis) can help determine the location of thrombosis and/or iliac vein compression. Treatment involves thrombolysis and stenting [22]. Therefore it can be concluded that the duplication and obstruction of the CIV in this patient may have contributed, alongside her coagulopathies and MTS, to edema, DVT, and chronic venous insufficiency of the LLE.

## Conclusions

We presented a case of a patient with significant edema of the LLE who was found to have a duplicated left CIV on venography. Understanding and being aware of the possible anomalies in pelvic venous anatomy are crucial in performing surgeries and carrying out interventional procedures. It is also essential to understand the associated pathologies of these anomalies and the symptoms they may directly cause.
